# *Deinagkistrodon acutus* envenomation: a report of three cases

**DOI:** 10.1186/s40409-017-0111-1

**Published:** 2017-03-23

**Authors:** Chin-Lung Cheng, Yan-Chiao Mao, Po-Yu Liu, Liao-Chun Chiang, Shu-Chen Liao, Chen-Chang Yang

**Affiliations:** 1 Department of Emergency Medicine, Kaohsiung Armed Forces General Hospital, Taipei, Taiwan; 2 Department of Emergency Medicine, Division of Clinical Toxicology, Taichung Veterans General Hospital, Taipei, Taiwan; 3 Department of Medicine, Division of Clinical Toxicology and Occupational Medicine, Taipei Veterans General Hospital, 201 Sec. 2, Shipai Road., Taipei, 112 Taiwan; 4 Institute of Environmental and Occupational Health Sciences, School of Medicine, National Yang-Ming University, Taipei, Taiwan; 5 Department of Medicine, Division of Infection, Taichung Veterans General Hospital, Taichung, Taiwan; 6 National Tsing Hua University, College of Life Sciences, Hsinchu, Taiwan; 7 National Health Research Institutes, National Institute of Infectious Diseases and Vaccinology, Zhunan, Miaoli Taiwan; 8 Department of Emergency Medicine, Chang Guang Memorial Hospital, Taipei, Taiwan

**Keywords:** Coagulopathy, Thrombocytopenia, Envenomation, *Deinagkistrodon acutus*, Snakebite

## Abstract

**Background:**

*Deinagkistrodon acutus* envenomation is associated with severe hematological and wound complications but is rarely described.

**Case presentation:**

Herein, we report three cases of victims bitten by *D. acutus* and indicate that rapid-onset severe coagulopathy and thrombocytopenia are distinct features of *D. acutus* snakebite, which are not observed in other crotaline snakebites (i.e., *Trimeresurus stejnegeri* and *Protobothrops mucrosquamatus*) in Taiwan. The toxic effects could occur as early as 2 to 3 h following *D. acutus* envenomation and persist if the administration of specific antivenom is delayed or even not commenced. Based on our findings, 2 to 4 vials of specific antivenom as the first dose should be administered to victims and repeated at 6 to 8 h intervals if coagulopathy or thrombocytopenia persists. Fresh frozen plasma or platelet replacement is probably safe as an adjunct therapy for *D. acutus* bite in the presence of venom-induced consumptive coagulopathy.

**Conclusion:**

Severe coagulopathy and thrombocytopenia could occur as early as 2 to 3 h after *D. acutus* envenomation. The current recommendation for antivenom is 2 to 4 vials as the first dose and repeated every 6– to 8 h if coagulopathy or thrombocytopenia persists. These cases studied may be helpful to first-line medical personnel in the early diagnosis and management of *D. acutus* envenomation among other crotaline snakebites in Taiwan.

## Background

Envenomation caused by snakebite comprises a worldwide public health problem, especially in Asia [1]. The venoms of snakes are a fascinating mix that allow the design of new drugs for use in medicine, as well as being a challenge for researchers in the development of specific antivenoms [2–5]. In Taiwan, six major venomous snakes are found, namely: *Trimeresurus stejnegeri*, *Protobothrops mucrosquamatus*, *Deinagkistrodon acutus* and *Daboia siamensis* in the family Viperidae, and *Naja atra* and *Bungarus multicinctus* in the family Elapidae [6]. *D. acutus* – also known as the hundred pacer, five pacer or Chinese moccasin – is the largest snake (80–155 cm) of the subfamily Crotalinae on the island [6]. It is additionally distributed throughout south China, Vietnam and possibly Laos [7]. *D. acutus* envenomation is rare; however, it is considered the most lethal and can result in life- or limb-threatening complications after envenomation [6, 8]. Although severe coagulopathy and thrombocytopenia, defined as an international normalized ratio (INR) of prothrombin time (PT) > 9 and platelet count < 50,000/mm^3^, are considered the hallmarks of *D. acutus* envenomation, little is known about the timing of onset and treatment with specific antivenom [9–13]. In the present study, we summarize the clinical manifestations and treatment of three cases of *D. acutus* envenomation admitted to Taichung Veterans General Hospital (VGH-TC) with the aim of improving the diagnosis and management of *D. acutus* envenomation.

## Case Presentation

### Case 1

A 36-year-old previously healthy man was bitten on his right hand by a snake during cleaning work around his home. He was immediately sent to a local hospital, with the dead snake identified as *D. acutus*. However, only two vials of antivenom for *T. stejnegeri* and *P. mucrosquamatus* were administered, because specific antivenom for *D. acutus* was not available and because the treating physician believed that cross-neutralization would occur. He received right upper limb fasciotomy on day 2 for suspected compartment syndrome. On day 3, he was transferred to VGH-TC due to worsening of his general condition and bleeding tendency. On arrival, the patient’s blood pressure (BP) was 109/47 mmHg, pulse 119/min, respiratory rate 20/min, and body temperature 38.5 °C.

Physical examination revealed continuous oozing from the wound and venous catheter insertion site, multiple hemorrhagic bullae, swelling extending up to the shoulder and gross hematuria in the urinary bag. Laboratory examination revealed a hemoglobin level of 5.8 g/dL (reference range 14–18 g/dL), platelet count 17,000/mm^3^ (reference range 150,000–400,000/mm^3^), fibrinogen 130 mg/dL (reference range 200–400 mg/dL), D-dimer > 1 μg/mL (reference range < 0.55 μg/mL), fibrinogen degradation products (FDPs) > 40 μg/mL (reference range < 10 μg/mL), and incoagulable blood [PT > 169 s; activated partial thromboplastin time (aPTT) > 224 s] (Table 1).

**Table 1:** Initial blood laboratory data of the three patients at Taichung Veterans General Hospital

Laboratory data (On arrival, day 1)	Case 1	Case 2	Case 3
White blood cell count (4,500–11,000/mm^3^)	4,700	12,300	9,300
Differential count (%)			
Neutrophil	76.9	84.7	44
Lymphocyte	15.2	7.6	45.2
Hemoglobin (male: 14–18, female 12–16 g/dL)	5.8	13.1	15.4
Platelet count (150,000–400,000/mm^3^)	17,000	14,000	17,000
Sodium (mEq/L)	136	142	144
Potassium (mEq/L)	4.0	4.0	3.7
Blood urea nitrogen (5–25 mg/dL)	12	14	24
Creatinine (0.7–1.4 mg/dL)	0.7	1.4	0.9
Alanine aminotransferase (male: 10–50, female: 10–35 U/L)	28	26	21
Aspartate aminotransferase (8–38 U/L)	30	43	26
Total bilirubin (0.2–1.6 mg/dL)	1.3	0.8	–
Direct bilirubin (0–0.3 mg/dL)	0	0.2	–
Lactate dehydrogenase (120–240 U/L)	206	181	242
Creatine kinase (10–160 U/L)	855	745	155
Prothrombin time (seconds)	>169	>164	>164
Activated partial thromboplastin time (s)	>224	>224	>224
Fibrinogen (200–400 mg/dL)	130	–	149.1
D-dimer (<0.55 μg/mL)	>1	–	–
Fibrinogen degradation products (FDPs < 10 μg/mL)	>40	–	>40

**Fig. 1:**
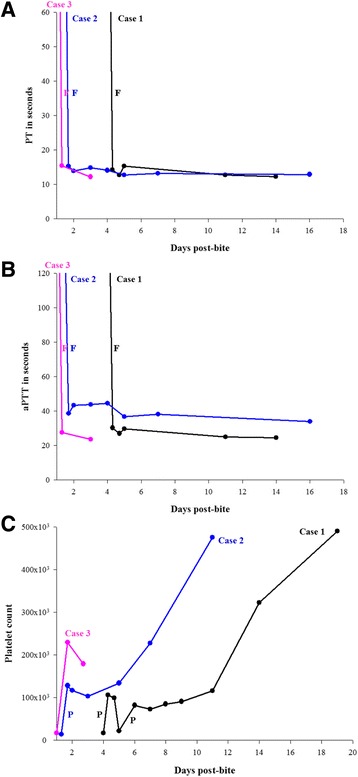
**a** Trend of PT in three patients. **b** Trend of aPTT in three patients. **c** Trend of platelet level in three patients. F: fresh frozen plasma transfusion. P: platelet concentrate transfusion

Three vials of monovalent antivenom for *D. acutus* and blood components [600 mL of packed red blood cells, 600 mL of fresh frozen plasma (FFP), and 300 mL of platelet concentrate] were immediately administered, resulting in a good response (Fig. 1). The patient’s blood coagulation normalized at 6 h after administration of the antivenom and oozing from the wound stopped. A follow-up coagulation profile did not reveal recurrent coagulopathy on days 4, 10 and 13. He had intermittent fever, and extensive wound necrosis that required repetitive debridement on days 8, 12 and 17 during hospitalization. Deep tissue cultures obtained during surgery grew *Pseudomonas aeruginosa*, *Morganella morganii*, *Staphylococcus aureus* and *Enterococcus* spp. Although thrombocytopenia persisted throughout days 4 to 10 (22,000–116,000/mm^3^), it was amenable to platelet transfusion and medical treatment. The bite wound improved gradually after antibiotic therapy and debridement. Staged wound closure was performed 3 weeks post-bite, and he was transferred to another hospital for a rehabilitation program 1 month later.

### Case 2

A 41-year-old previously healthy woman was bitten on the left ankle by a snake while she collected herbs in northwestern Taiwan. She was sent to a local hospital 3 h later, where thrombocytopenia (3000/mm^3^) and incoagulable blood (PT > 100 s) were noted. The snake was identified as *D. acutus* by the patient through a picture; however, two vials of antivenom for *T. stejnegeri* and *P. mucrosquamatus* were administered for an unknown reason. She was then referred to VGH-TC 7.5 h post-bite.

On arrival, her BP was 172/94 mmHg, pulse 108/min, respiratory rate 20/min, and body temperature 39.3 °C. Physical examination revealed many hemorrhagic bullae scattered along the calf, continuous oozing from the fang marks, and painful swelling extending to the knee region. Laboratory examination disclosed thrombocytopenia (14,000/mm^3^) and PT of > 169 s and aPTT of > 224 s. Four vials of monovalent antivenom for *D. acutus* and blood components (200 mL of FFP and 300 mL of platelet concentrate) were administered at 8.5 h post-bite. Her coagulation profiles normalized at 15 h post-bite. On day 2, her platelet count increased to 158,000/mm^3^. Due to prolonged aPTT (43.3–44.6 s), another seven vials of antivenom (2 to 3 vials every 6 to 8 h) were sequentially administered without measurable responses.

The leg wound was complicated with necrotizing fasciitis that required repetitive debridement on days 21 and 31 and supplemental hyperbaric oxygen therapy. The wound cultures obtained during surgery revealed *S. aureus*, *Enterococcus* spp. and *Bacteroides fragilis*. After antibiotic treatment, her wound infection improved and split-thickness skin grafting was performed after debridement on day 31. The patient was discharged on day 47 post-bite with good functional recovery of the leg.

### Case 3

A 69-year-old previously healthy woman was bitten on the left middle finger by a snake while collecting firewood. Painful swelling, tissue ecchymosis, and oozing from the wound developed a few minutes later. The patient was sent to VGH-TC 2 h post-bite. The dead snake brought in by the patient was identified as *D. acutus*. On arrival, her BP was 200/120 mmHg, pulse 95/min, respiratory rate 22/min, and body temperature 36.7 °C. Physical examination revealed swelling of left hand. A low platelet count (17,000/mm^3^), PT > 169 s, aPTT > 224 s, fibrinogen level 149.1 mg/dL and FDPs > 40 μg/mL were noted in laboratory analyses.

Four vials of monovalent antivenom for *D. acutus* and 1000 mL of FFP were administered. Seven hours post-bite, both PT and aPTT normalized, and the platelet count increased to 230,000/mm^3^. Because of recurrent oozing from the wound, another four vials of antivenom (two at 12 h intervals) were administered without examination of PT and aPTT levels in the following 24 h. Although the surgeon recommended partial finger amputation due to finger necrosis, the patient declined and insisted on being discharged against medical advice on day 5. No recurrent coagulopathy was noted prior to discharge; however, the patient did not return for follow-up.

## Discussion

*T. stejnegeri* and *P. mucrosquamatus* account for more than 70% of snakebite cases each year in Taiwan, which share similar clinical manifestations as well as the same treatment with bivalent specific antivenom [6, 8]. Sporadic cases with INR above 1.67 and platelet counts of 36,000/mm^3^ were reported in *P. mucrosquamatus* envenomation, and none manifested systemic bleeding [11, 14]. In contrast, severe coagulopathy and thrombocytopenia are the main laboratory findings of *D. acutus* envenomation in addition to serious wound complications and systemic bleeding [11–13]. Valenta et al. [15] reported a victim of *D. acutus* bite who developed incoagulable blood between 1.5 and 7 h post-bite. Hung et al. [13] reported the case of a man with persistent thrombocytopenia 44 h after envenomation; his platelet level was normal 30 min post-bite. In our observation, rapid-onset and severe coagulopathy and thrombocytopenia developed as early as 2 to 3 h post-bite may persist if correct antivenom is not administered.

*D. acutus* venom is composed of several hemotoxins, including the thrombin-like enzymes (TLEs), anticoagulant toxins, platelet aggregation inhibitors, hemorrhagins, and enzymes that facilitate venom spreading [16–19]. The anticoagulation effect of TLEs occurs rapidly, and circulating fibrinogen levels start to fall within 30 min, reaching 9% of the normal value within 2 h [16]. Anticoagulant toxins inactivate prothrombin, tissue factor and coagulation factors V and IX/X, resulting in a transient but marked prolongation of blood coagulation within 5 min after injection [16, 20, 21]. The platelet inhibitors, mainly adenosine diphosphatase, inhibit platelet aggregation in the presence of adenosine diphosphate or collagen [17]. The hemorrhagins (e.g., snake venom metalloproteinases) cause extensive vascular damage and increase vascular permeability [22]. These effects may explain the remarkable bleeding tendency and wound complications in *D. acutus* envenomation.

The Taiwan government produces four types of antivenom against the six major venomous snakebites [8]. Concerning the monovalent antivenom for *D. acutus*, each vial roughly neutralizes 52 mg of venom. The Taiwan Poison Control Center thus recommends that 2 to 4 vials of antivenom be administered in a envenomed patient because the mean amount of *D. acutus* venom injected is 105.1 mg [8]. However, this recommendation was not validated and there is no standard dosing regimen available in Taiwan. According to our findings, the current recommendation is 2 to 4 vials of antivenom to be administered as the first dose and repeated at 6 to 8 h intervals if coagulopathy or thrombocytopenia persists (e.g., INR > 3, aPTT > 50s, and platelet level < 20,000/mm^3^) [23, 24]. Although cross-neutralization occurs between bivalent antivenom for *T. stejnegeri* and *P. mucrosquamatus* and monovalent antivenom for *D. acutus*, substitution should be avoided as the bivalent antivenom has only a weak cross-reactivity with the *D. acutus* venom [13].

All of our cases had a rapid improvement in coagulopathy after receiving specific antivenom. In case 2, s slightly prolonged aPTT persisted for 4 days without clinical thrombosis or bleeding tendency. Although a workup for prolonged aPTT was not performed in the case, it was probably unrelated to the venom because no apparent response was observed after repetitive antivenom administration [25]. In case 1, thrombocytopenia did not show improvement after antivenom therapy, which was probably attributable to uncontrolled infection. Nevertheless, Valenta et al. [15] reported a single case of *D. acutus* envenomation who manifested severe coagulopathy in the absence of thrombocytopenia. Therefore, the exact mechanism of thrombocytopenia in *D. acutus* snakebite remains unclear.

Evidence suggests that, when compared with antivenom alone, early FFP replacement therapy shortens coagulopathy in patients suffering from hemotoxic snake envenomation and, theoretically, lower the risk of major bleeding [26]. The bleeding risk may be pronounced in cases of severe thrombocytopenia [10]. However, in cases of venom-induced thrombotic microangiopathy, platelet transfusion may be problematic [27]. In our study, no elevation of blood bilirubin or lactate dehydrogenase was found, and the first two cases had received platelet concentrate transfusions, resulting in good responses. It appears to be safe to use the blood component therapy during *D. acutus* envenomation. In case 3, the platelet count spontaneously normalized after 24 h with FFP and specific antivenom administration alone. This effect may have occurred because of the relatively modest injury in this case. However, the patient received a higher dosage (eight vials) of antivenom, which was sufficient to counteract the venom effects on platelets.

## Conclusion

Severe coagulopathy and thrombocytopenia could occur as early as 2 to 3 h after *D. acutus* envenomation. The current recommendation for antivenom therapy is 2 to 4 vials as the first dose and repeat it every 6 to 8 h if coagulopathy or thrombocytopenia persists. Our observation may be helpful to first-line medical personnel in the timely diagnosis and management of *D. acutus* bites.

## Abbreviations

aPTT: activated partial thromboplastin time
BP: Blood pressure
FDPs: Fibrinogen degradation products
FFP: Fresh frozen plasma
INR: International normalized ratio
PT: Prothrombin time
TLEs: Thrombin-like enzymes
VGH-TC: Taichung Veterans General Hospital
